# Breastfeeding history and the risk of overweight and obesity in middle-aged women

**DOI:** 10.1186/s12905-021-01332-2

**Published:** 2021-05-11

**Authors:** Elżbieta Cieśla, Ewa Stochmal, Stanisław Głuszek, Edyta Suliga

**Affiliations:** 1 Institute of Health Sciences, Medical College, Jan Kochanowski University, Kielce, Poland; 2 Institute of Medical Sciences, Medical College, Jan Kochanowski University, Kielce, Poland

**Keywords:** Lactation, Parity, Menopausal status

## Abstract

**Background:**

The increased metabolic activity required to sustain breastfeeding and its associated milk production helps to reduce maternal fat stores accumulated during pregnancy. This study aims to assess the association between breastfeeding duration and fatness indices in middle-aged women.

**Methods:**

The analysis was carried out in a group of 7500 parous 55.5 ± 5.3 year old women included body mass index, body fat percentage, and waist-to-height ratio. The likelihood of excessive weight or obesity in relation to total breastfeeding time using multivariate logistic regression analyses.

**Results:**

An analysis of adjusted odds ratios did not show significant associations between breastfeeding duration and the risk of excessive weight and obesity in premenopausal women. After menopause, women who gave birth to 2 children and breastfed 1–6 and > 12 months had a lower risk of abdominal obesity (OR 0.70; 95% CI 0.50–0.99; *p* = 0.042; and OR 0.68; 95% CI 0.47–0.98; *p* = 0.039). Women who gave birth to 3 or more children and breastfed for 1–6 months, also showed a lower risk of overweight (OR 0.52; 95% CI 0.27–0.99; *p* = 0.047), compared to those ones that have never breastfed. There was no relationship found between the duration of lactation and the risk of excessive body fat.

**Conclusion:**

Breastfeeding may have some beneficial, long-term effect on the risk of excessive weight and abdominal obesity in women.

**Supplementary Information:**

The online version contains supplementary material available at 10.1186/s12905-021-01332-2.

## Background

Excessive body fat is a significant risk factor for many diseases including type 2 diabetes, cardiovascular disease, and cancer [1–6]. Epidemiological data indicate that obesity is more common in women than in men [7, 8]. Female-specific factors related to the risk of obesity include the duration of lactation, parity, and menopausal status. Pregnant women have increased energy demands associated with the development and growth of the fetus, placenta, enlargement of the uterus, mammary glands, and an increased in blood volume. During pregnancy, the female body also prepares for breastfeeding by storing nutrients and energy needed for milk production. During this period, excessive energy intake in relation to needs, may increase the body's fatty tissue, making it difficult to return to the pre-pregnancy weight, and maintain normal body mass over a longer period of time [9, 10].

The increased metabolic activity required to sustain breastfeeding and its associated milk production helps to reduce maternal fat stores accumulated during pregnancy [11]. It has been estimated that breastfeeding mothers need about 2.8 megajoules (MJ) (670 kcal) of additional energy per day to produce milk, of which about 2.1 MJ (500 kcal) should be acquired from food; the rest should come from fat stores accumulated during pregnancy [12]. Breastfeeding can therefore have a beneficial effect on the body fat index in women. From 8 retrospective studies a positive association between breastfeeding and weight changes was noted in only 2 papers, whereas 14 of 35 prospective studies in which women were evaluated for weight ≤ 2 years after delivery, found a beneficial effect of breastfeeding on body weight changes. The remaining 21 studies did not show such relationships [13]. Of the 5 studies that were considered to be of high methodological quality, 4 showed a positive association between breastfeeding and weight change. McClure et al. found that 7 years post-partum, the amount of visceral fat measured by computed tomography was greater among those women who breastfed for less than 3 months after the birth of each child, compared to those who breastfed longer [14]; however, they did not observe such a relation regarding body mass index (BMI) and other fatness indicators. Bobrow et al. showed that postmenopausal women who had breastfed, had a significantly lower BMI than those who had never breastfed [15]. Snyder et al. found that breastfeeding was significantly associated with smaller waist circumference after a mean follow-up period of 11 (7–15) years [16]. Results of a cluster-randomized controlled trial did not confirm that a longer time spent breastfeeding led to any significant reduction in fatty tissue more than 11 years postpartum [17]. Studies on the relationship between breastfeeding and long-term weight changes in women are inconclusive. It is therefore important to examine breastfeeding history as an independent factor that can potentially have a long-term influence on female fatness.

Most studies have confirmed that parity strongly increases the risk of excess body mass in women whilst extended breastfeeding duration has the opposite effect i.e. lower body mass [15, 18, 19]. Weight gain in women who have given birth is much greater than in those who have never given birth [18, 19]. Many studies show that menopausal transformation in women leads to an increased risk of obesity [20–24]. Menopause onset is associated with decreased energy expenditure and fat oxidation which can predispose to excess of body fat mass [20, 24].

The aim of the study was to assess the relationship between breastfeeding duration and body fat indices: BMI, body fat percentage (%BF) and waist-to-height ratio (WHtR) in middle-aged women. All analyzes were carried out separately for premenopausal and postmenopausal women, in three fertility categories.

## Methods

### Design and sample

The research material consists of the data of 8725 participants of the PONS (POlish-Norwegian Study) project, carried out 2010–2012, in the province of Świętokrzyskie in Poland. Ethics Committee from the Cancer Centre and Institute of Oncology in Warsaw, No. 69/2009/1/2011 (data collection), and Committee on Bioethics at the Faculty of Health Sciences, Jan Kochanowski University in Kielce, Poland (No. 29/2015) (data analysis) approved the study.

This was a secondary data analysis aiming to assess the health status of the female adult population of the Świętokrzyskie region in Poland. Anthropometric and blood pressure measurements were performed, and body composition analysis and biochemical blood analysis were carried out. An extensive questionnaire interview was conducted to gather socio-economic data, lifestyle information, and medical history. The study protocol is described in detail in previously published articles [25–27].

The analysis excluded 327 women with diagnosed cancer, 263 with incomplete data and 635 women who had never given birth. The final sample consisted an ethnically-homogenous, Caucasian population of 7500 parous women (Additional file 1: Fig. SM1).

### Measurements

All measurements were obtained by trained nurses. The following data was used in this study: anthropometric measurements of body weight, height and waist circumference, from which BMI (kg/m^2^) and WHtR (waist circumference/height) were calculated, as well as body fat percentage (% BF) measurement using the bioelectrical impedance method.

### Sociodemographic and lifestyle data

The questionnaire interview was conducted by trained nurses. The following socio-demographic and lifestyle information was collected in the survey: age (years), education (years), place of residence (urban; rural), marital status (married or in a stable relationship; single or widower), parity (1, 2, 3 and more children), menopausal status (premenopausal, postmenopausal), total duration of breastfeeding in months (then divided into breastfed 1–6; 7–12; > 12 months or never breastfed) and the use of hormone therapy (ever, never). The subjects were also divided into never, former or current smokers. The long form International Physical Activity Questionnaire (IPAQ) was used to assess physical activity, and it was expressed as Moderate-to-Vigorous (MVPA) and light physical activity (LPA) (Metabolic Equivalent of Task—MET/min/day^−1^) [28].

Sitting time was expressed in minutes/day. Food consumption data was collected using the Food Frequency Questionnaire (FFQ), based on a previously-developed and validated FFQ for the Polish branch of the PURE study [29]. As it was not possible to calculate the calorific value of the subjects’ diets based on the collected data, the analysis included 3 groups of products that could potentially have the greatest impact on fatness: fats (butter, lard, margarine, mayonnaise, rapeseed oil, soya, sunflower oil, olive oil, and other oils), sweets (sugar, chocolate and chocolate-products, candies, cakes, cookies), whole grains (whole grain bread, groats, cereals). Answers related to the consumption frequency of products with standard portion sizes, and were transformed into daily consumption doses and standardized by z-score.

### Definition of terms

The overweight group included women with BMI ≥ 25.0 kg/m^2^, it was assumed that excess fatty tissue indicating obesity was > 35% BF [30], and for abdominal obesity it was WHtR ≥ 0.5 [31]. The North American Menopause Society, postmenopausal women include those with amenorrhea for at least 12 months [32].

### Statistical analysis

All categorical variables were expressed as frequency and percentage (n, %) and all continuous variables were reported as means and standard deviations (X ± SD). Differences in baseline characteristics between normal and abnormal adiposity were assessed using the Mann–Whitney U test for continuous variables and the Chi-square test for categorical variables. The probability of excessive weight and/or obesity in relation to total breastfeeding duration was assessed using multivariate logistic regression analyses, calculating the odds ratios (ORs) and 95% confidence intervals (CIs). The analyses were carried out separately in three categories of parity, in raw and adjusted models for confounding variables. The control group (ref.) consisted of women who had never breastfed. In models adjusted as confounders, the following were adopted: age; years of education, MVPA, LPA, sitting time, consumption of fats, sweets, whole grains, (continuous variables), and place of living (ref. urban), marital status (ref. single), smoking (ref. never smokers), and hormone therapy (ref. never). Confounders were chosen based on relationships found in previous analyses of the collected data [23, 26, 33, 34] as well as literature analysis [13]. Analyses were performed using the statistical package Statistica 13.3 (TIBCO SOFTWARE INC, Polish version, PL, Cracow). A *p* value ≤ 0.05 represented statistical significance.

## Results

The average age of the women studied was 55.5 ± 5.3 years. In the premenopausal group, overweight and obese women were older, less well-educated, inhabited rural areas more than urban, and were more often in stable relationships compared to those with normal weight (Table 1). They also devoted less time to LPA, and declared a lower consumption of sugar and sweets. Women with more body fat (%BF > 35%) more often declared limiting fat intake. Women with abdominal obesity (WHtR ≥ 0.5) consumed sweets with a similar frequency as women without abdominal obesity, while declaring a lower intake of whole grains. In the compared groups (BMI ≥ 25 kg/m^2^ vs. < 25 kg/m^2^, %BF > 35% vs. ≤ 35%, and WHtR ≥ 0.5 vs. < 0.5), no significant differences were found in the time spent in MVPA or sitting time. The percentage of smokers was similar in the compared groups. Overweight and obese women more often gave birth to 3 or more children and less often used hormonal therapy. The percentage of women who breastfed for > 12 months was higher in the overweight group (BMI ≥ 25 kg/m^2^) than in the normal weight group. Differences between the compared groups were not statistically significant for the other obesity indices.

**Table 1:** Characteristics of premenopausal participants included in these analyses (N = 2048)

Variables	BMI			%BF			WHtR		
	< 25 kg/m^2^	≥ 25 kg/m^2^	*p*	≤ 35%	> 35%	*p*	< 0.5	≥ 0.5	*p*
	n = 815	n = 1233		n = 1094	n = 954		n = 803	n = 1245	
Age, X ± SD	48.98 ± 3.02	50.02 ± 3.22	< 0.001^a^	49.22 ± 3.08	50.05 ± 3.23	< 0.001^a^	48.92 (3.02)	50.05 (3.20)	< 0.001^a^
Years of education, X ± SD	14.68 ± 3.06	13.80 ± 3.15	< 0.001^a^	14.51 ± 3.11	13.73 ± 3.13	< 0.001^a^	14.92 (2.97)	13.65 (3.15)	< 0.001^a^
Place of living, N (%)									
City	517 (63.44)	663 (53.77)	< 0.001^b^	670 (61.24)	510 (53.46)	< 0.001^b^	541 (67.37)	639 (51.33)	< 0.001^b^
Village	298 (36.56)	570 (46.23)		424 (38.76)	444 (46.54)		262 (32.63)	606 (48.67)	
Marital status, N (%)									
Single	159 (19.51)	177 (14.36)	0.002^b^	197 (18.01)	139 (14.57)	0.036^b^	151 (18.80)	185 (14.86)	0.019^b^
Married	656 (80.49)	1056 (85.64)		897 (81.99)	815 (85.43)		652 (81.20)	1060 (85.14)	
Physical activity (MET/min/day^−1^) X ± SD									
Moderate-to-Vigorous	495.57 ± 499.05	510.56 ± 500.87	0.352^a^	505.11 ± 503.18	504.00 ± 496.76	0.686^a^	487.00 ± 484.55	515.94 ± 509.71	0.253^a^
Light	253.82 ± 228.54	237.84 ± 228.99	0.050^a^	253.22 ± 228.92	233.85 ± 228.53	0.020^a^	257.65 ± 234.57	235.52 ± 224.82	0.019 ^a^
Sitting time (min/day) X ± SD	307.94 ± 139.99	310.84 ± 141.81	0.747^a^	307.19 ± 140.02	312.55 ± 142.26	0.426^a^	316.44 ± 141.92	305.33 ± 140.39	0.055^a^
Fats (servings/day) X ± SD	2.87 ± 1.94	2.76 ± 1.99	0.071^a^	2.87 ± 1.94	2.73 ± 2.00	0.044^a^	2.82 ± 1.92	2.79 ± 2.00	0.399 ^a^
Sweets (servings/day) X ± SD	3.62 ± 3.27	3.20 ± 3.01	0.004^a^	3.54 ± 3.17	3.17 ± 3.05	0.004^a^	3.37 ± 3.18	3.36 ± 3.08	0.898 ^a^
Whole grains (servings/day) X ± SD	2.01 ± 2.18	1.86 ± 2.01	0.526^a^	1.93 ± 2.14	1.90 ± 2.01	0.370^a^	2.12 ± 2.19	1.79 ± 2.00	0.001^a^
Smoking, N (%)									
Current smokers	174 (21.35)	219 (17.76)	0.026^b^	212 (19.38)	181 (18.97)	0.253^b^	161 (20.05)	232 (18.63)	0.602^b^
Never smokers	462 (56.69)	699 (56.69)		634 (57.95)	527 (55.24)		456 (56.79)	705 (56.63)	
Former smokers	179 (21.96)	315 (25.55)		248 (22.67)	246 (25.79)		186 (23.16)	308 (24.74)	
Hormone therapy N (%)									
Yes	195 (23.93)	221 (17.92)	0.001^b^	248 (22.67)	168 (17.61)	0.004^b^	209 (26.03)	207 (16.63)	< 0.001^b^
No	620 (76.07)	1012 (82.08)		846 (77.33)	786 (82.39)		594 (73.97)	1038 (83.37)	
Parity, N (%)									
1	173 (21.23)	190 (15.41)	< 0.001^b^	212 (19.38)	151 (15.83)	0.011^b^	187 (23.29)	176 (14.14)	< 0.001^b^
2	440 (53.99)	606 (49.15)		569 (52.01)	477 (50.00)		433 (53.92)	613 (49.24)	
3 and more	202 (24.79)	437 (35.44)		313 (28.61)	326 (34.1734,17)		183 (22.79)	456 (36.63)	
Breastfeeding status, N (%)									
Never breastfed	79 (8.69)	121 (9.81)	0.059^b^	102 (9.32)	98 (10.27)	0.371^b^	80 (9.96)	120 (9.64)	0.153 ^b^
Breastfed 1–6 months	264 (32.39)	337 (27.33)		333 (30.44)	268 (28.09)		256 (31.88)	345 (27.71)	
7–12 months	187 (22.94)	283 (22.95)		259 (16.26)	211 (22.12)		183 (22.79)	287 (23.05)	
> 12 months	285 (34.97)	492 (39.90)		400 (36.56)	377 (39.52)		284 (35.37)	493 (39.60)	

BMI, body mass index; %BF, body fat percentage; WHtR, waist-to-hight ratio; X, arithmetic mean; SD, standard deviation

^a^Mann-Whitney U test

^b^Chi-square test

Among postmenopausal women, overweight and obese women were also older, less well-educated and inhabited rural areas more often than urban, compared to women with normal weight (Table 2). Marital status was not a significant factor in the occurrence of excessive weight and abdominal obesity, though participants in stable relationships had a higher body mass (%BF > 35%). Overweight and obese women spent less time in LPA, smoked less often, used hormone therapy less often, and declared lower sugar and sweets consumption, while women with abdominal obesity (WHtR ≥ 0.5) also declared a lower consumption of whole grains. There were no significant differences in fat intake or sitting time between groups. The percentage of overweight women who gave birth to 3 or more children was higher, whereas the percentage who gave birth to only 1 child was lower. A higher percentage breastfed > 12 months, and a lower percentage breastfed 1–6 months.

**Table 2:** Characteristics of postmenopausal participants included in these analyses (N = 5452)

Variables	BMI			%BF			WHtR		
	< 25 kg/m^2^	≥ 25 kg/m^2^	p	≤ 35%	> 35%	p	< 0.5	≥ 0.5	p
	n = 1400	n = 4052		n = 1909	n = 3543		n = 1118	n = 4334	
Age X ± SD	56.70 ± 4.24	58.05 ± 4.07	< 0.001^a^	56.91 ± 4.25	58.13 ± 4.04	< 0.001^a^	56.52 ± 4.21	58.01 ± 4.08	< 0.001^a^
Years of education X ± SD	13.46 ± 2.97	12.51 ± 3.08	< 0.001^a^	13.25 ± 3.03	12.48 ± 3.07	< 0.001^a^	13.83 ± 2.95	12.48 ± 3.05	< 0.001^a^
Place of living N (%)									
City	993 (70.93)	2442 (60.27)	< 0.001^b^	1312 (68.73)	2123 (59.92)	< 0.001^b^	831 (74.33)	2604 (60.08)	< 0.001^b^
Village	407 (29.07)	1610 (39.73)		597 (31.27)	1420 (40.08)		287 (25.67)	1730 (39.92)	
Marital status N (%)									
Single	330 (23.57)	988 (24.38)	0.541^b^	496 (25.98)	1318 (23.20)	< 0.001^b^	263 (23.52)	1055 (24.34)	0.569^b^
Married	1070 (76.43)	3064 (75.62)		1413 (74.02)	2721 (76.80)		855 (76.48)	3279 (75.66)	
Physical activity (MET/min/day^−1^) X ± SD									
Moderate-to-Vigorous	408.50 ± 427.56	435.74 ± 459.93	0.055^a^	417.58 ± 431.55	434.76 ± 462.53	0.160^a^	416.34 ± 434.02	431.94 ± 456.46	0.352 ^a^
Light	221.99 ± 207.32	201.42 ± 202.35	< 0.001^a^	225.63 ± 215.17	196.51 ± 196.70	< 0.001 ^a^	236.60 ± 220.01	199.00 ± 198.72	< 0.001^a^
Sitting time (min/day) X ± SD	287.17 ± 134.84	284.69 ± 128.72	0.968^a^	284.89 ± 135.31	285.57 ± 127.56	0.411^a^	291.48 ± 138.47	283.74 ± 128.09	0.260 ^a^
Fats (servings/day) X ± SD	2.62 ± 1.89	2.71 ± 1.94	0.414^a^	2.62 ± 1.90	2.73 ± 1.94	0.153^a^	2.58 ± 1.89	2.72 ± 1.93	0.177^a^
Sweets (servings/day) X ± SD	3.57 ± 4.32	2.90 ± 2.96	< 0.001^a^	3.36 ± 4.01	2.91 ± 2.97	< 0.001^a^	3.30 ± 4.35	3.01 ± 3.07	0.030^a^
Whole grains (servings/day) X ± SD	1.91 ± 2.00	1.98 ± 2.05	0.175^a^	1.96 ± 2.06	1.97 ± 2.03	0.879^a^	2.11 ± 2.14	1.93 ± 2.01	0.021^a^
Smoking N (%)									
Current smokers	393 (28.07)	590 (14.56)	< 0.001^b^	485 (24.41)	498 (14.06)	< 0.001^b^	286 (25.58)	697 (16.08)	< 0.001^b^
Never smokers	617 (44.07)	2175 (53.68)		887 (46.46)	1905 (53.77)		506 (45.26)	2286 (52.75)	
Former smokers	390 (27.86)	1287 (31.76)		537 (28.13)	1140 (32.18)		326 (29.16)	1351 (31.17)	
Hormone therapy N(%)									
Yes	186 (13.29)	351 (8.66)	< 0.001^b^	238 (12.47)	299 (8.44)	< 0.001^b^	170 (15.21)	367 (8.47)	< 0.001^b^
No	1214 (86.71)	3701 (91.34)		1671 (87.53)	3244 (91.56)		948 (84.79)	3967 (91.53)	
Parity N (%)									
1	310 (22.14)	601 (14.83)	< 0.001^b^	411 (21.53)	500 (14.11)	< 0.001^b^	277 (24.78)	634 (14.63)	< 0.001^b^
2	720 (51.43)	2018 (49.80)		962 (50.39)	1776 (50.13)		578 (51.70)	2160 (49.84)	
3 and more	370 (26.43)	1433 (35.37)		536 (28.08)	1267 (35.76)		263 (23.52)	1540 (35.53)	
Breastfeeding status N (%)									
Never breastfed	169 (12.07)	474 (11.70)	< 0.001^b^	237 (12.41)	406 (11.46)	< 0.001^b^	140 (12.52)	503 (11.61)	< 0.001^b^
Breastfed 1–6 months	545 (12.07)	1305 (32.21)		726 (38.03)	1124 (31.72)		451 (40.34)	1399 (32.28)	
7–12 months	327 (23.36)	943 (23.27)		436 (22.84)	834 (23.54)		242 (21.65)	1028 (23.72)	
> 12 months	359 (25.64)	1330 (32.82)		510 (26.72)	1179 (33.28)		285 (25.49)	1404 (32.40)	

BMI, body mass index; %BF, body fat percentage; WHtR, waist-to-hight ratio; X, arithmetic mean; SD, standard deviation

^a^Mann-Whitney U test

^b^Chi-square test

An analysis of the unadjusted ORs in premenopausal women showed no significant association between the duration of lactation and the risk of excessive weight and obesity in any of the three parity categories (Additional file 2: Table SM1). In postmenopausal women who gave birth to 2 children, the risk of abdominal obesity was significantly lower in those who breastfed for 1–6, as well as > 12 months, compared to those who had never breastfed (Additional file 2: Table SM2). In the group who gave birth to 3 or more children, the risk of excessive weight (BMI ≥ 25 kg/m^2^) and abdominal obesity (WHtR ≥ 0.5), was significantly lower in those who breastfed for 1–6 months compared to non-breastfeeding mothers. A similar tendency was observed with longer lactation, but it was not statistically significant. There was no association between the duration of lactation and the risk of excessive body fat (%BF > 35%).

The analysis of adjusted ORs did not show any significant associations between the length of breastfeeding and the risk of excessive weight and obesity in premenopausal women of any parity (Table 3). The results for those in the breastfeeding group who gave birth to 3 or more children were 0.39 to 0.51 for BMI ≥ 25 kg/m^2^, and 0.54 to 0.76 for WHtR ≥ 0.5. Although not statistically significant, these values were similar and sometimes lower than in the postmenopausal group. Postmenopausal women who gave birth to one child and breastfed for 7–12 months had a higher risk of abdominal obesity compared to those who did not breastfeed (Table 4). Among women who gave birth to 2 children, breastfeeding was associated with a lower risk of abdominal obesity (WHtR ≥ 0.5). Participants who gave birth to 3 or more children and breastfed for 1–6 months had a lower risk of being overweight (BMI ≥ 25 kg/m^2^) compared to those who had never breastfed.

**Table 3:** Multivariable logistic regression analysis for overweight and obesity in relation to breastfeeding duration in premenopausal women (adjusted)

Parity	Breastfeeding status	BMI ≥ 25 kg/m^2^		%BF > 35%		WHtR ≥ 0.5	
		OR; 95%CI	*p*	OR; 95%CI	*p*	OR; 95%CI	*p*
1 child	Did not breastfeed	1.00		1.00		1.00	
	breastfeed 1–6 months	1.04; 0.61–1.79	0.880	0.95; 0.55–1.63	0.842	0.93; 0.54–1.59	0.787
	7–12 months	1.01; 0.49–2.09	0.971	0.77; 0.37–1.61	0.491	0.92; 0.45–1.89	0.821
	> 12 months	0.67; 0.30–1.51	0.331	0.46; 0.19–1.09	0.079	0.57; 0.25–1.29	0.176
2 children	Did not breastfeed	1.00		1.00		1.00	
	breastfeed 1–6 months	0.78; 0.48–1.29	0.334	0.75; 0.47–1.22	0.255	0.91; 0.55–1.51	0.721
	7–12 months	0.94; 0.57–1.55	0.800	0.80; 0.49–1.31	0.381	0.89; 0.54–1.49	0.666
	> 12 months	1.07; 0.65–1.78	0.780	0.98; 0.60–1.59	0.932	1.02; 0.61–1.71	0.922
3 and more children	Did not breastfeed	1.00		1.00		1.00	
	breastfeed 1–6 months	0.39; 0.12–1.19	0.099	0.93; 0.37–2.38	0.887	0.54; 0.17–1.70	0.294
	7–12 months	0.44; 0.15–1.31	0.142	0.88; 0.36–2.12	0.769	0.76; 0.25–2.27	0.622
	> 12 months	0.51; 0.18–1.45	0.210	1.07; 0.47–2.44	0.875	0.71; 0.25–2.00	0.515

BMI, body mass index; %BF, body fat percentage; WHtR, waist-to-hight ratio; OR, odds ratio; CI, confidence interval

**Table 4:** Multivariable logistic regression analysis for overweight and obesity in relation to breastfeeding duration in postmenopausal women (adjusted)

Parity	Breastfeeding status	BMI ≥ 25 kg/m^2^		%BF > 35%		WHtR ≥ 0.5	
		OR; 95%CI	p	OR; 95%CI	p	OR; 95%CI	p
1 child	Did not breastfeed	1.00		1.00		1.00	
	breastfeed 1–6 months	1.16; 0.83–1.63	0.708	1.01; 0.73–1.39	0.752	1.29; 0.91–1.82	0.157
	7–12 months	1.41; 0.86–2.33	0.411	1.33; 0.83–2.12	0.156	**1.78; 1.05–3.00**	**0.031**
	> 12 months	1.34; 0.69–2.60	0.693	0.89; 0.48–1.66	0.480	1.29; 0.66–2.53	0.461
2 children	Did not breastfeed	1.00		1.00		1.00	
	breastfeed 1–6 months	0.82; 0.60–1.12	0.433	0.95; 0.72–1.25	0.715	**0.70; 0.50–0.99**	**0.042**
	7–12 months	0.78; 0.56–1.08	0.171	0.94; 0.71–1.26	0.700	0.76; 0.53–1.09	0.139
	> 12 months	0.89; 0.63–1.24	0.171	1.03; 0.76–1.38	0.863	**0.68; 0.47–0.98**	**0.039**
3 and more children	Did not breastfeed	1.00		1.00		1.00	
	breastfeed 1–6 months	**0.52; 0.27–0.99**	**0.047**	0.75; 0.44–1.26	0.273	0.56; 0.27–1.13	0.105
	7–12 months	0.64; 0.34–1.20	0.160	0.89; 0.54–1.48	0.667	0.68; 0.34–1.38	0.288
	> 12 months	0.74; 0.41–1.36	0.335	1.05; 0.65–1.69	0.852	0.81; 0.42–1.58	0.538

BMI, body mass index; %BF, body fat percentage; WHtR, waist-to-hight ratio; OR, odds ratio; CI, confidence interval; bold indicate statistically significant results

## Discussion

This study showed that in postmenopausal women who had given birth to 2, 3 or more children, breastfeeding was associated with a lower risk of excessive weight and abdominal obesity, compared to women of the same parity who did not breastfeed at all. Adjustment for confounding factors did weaken the associations slightly, but they remained statistically significant in many groups.

Most studies confirm an inverse association between breastfeeding duration and the risk of obesity [15, 35–37]. In the Norwegian population, the probability of obesity in women under the age of 50, who had never breastfed, was 3.37 times higher compared to those who had breastfed for at least 24 months [38]. Bobrow et al. reported that in postmenopausal women, the mean BMI was 0.22 kg/m^2^ lower for each additional 6 months of breastfeeding [15].

Some studies, however, have not confirmed the association between the lactation duration and fatness [17, 39]. Sharma et al. found that women with obesity six years postpartum and who fully complied with the recommendations regarding breastfeeding at that time (i.e., they were exclusively breastfeeding for ≥ 4 months and continuing breastfeeding for ≥ 12 months) had a lower body mass than obese women who had never breastfed; they did not find such associations in women with normal weight or who were overweight [40].

The results of our study are largely consistent with those studies which demonstrate a positive effect of breastfeeding on reducing the risk of abdominal obesity. In a cross-sectional study of American women who were on average 7 years postpartum, the amount of visceral adipose tissue measured by computed tomography was over 20 cm^2^ higher among mothers who breastfed for < 3 months after the birth of each child, compared to those who breastfed longer [14, 36]. Women of diverse ethnicity (Filipino, Caucasian, and African-American) who breastfed > 3 months had lower volumes of visceral fat at age 55–80 years than those who breastfed ≤ 3 months [36]. These relationships in both studies were independent of covariates. Women who breastfed for > 6 months, had a significantly smaller waist circumferences 10 years postpartum than those who breastfed for a shorter period [16]. Kirkegaard et al. who showed a weak but significant inverse association between breastfeeding duration and body weight 7 years postpartum, and found this association to be much stronger in BMI-adjusted waist circumference analysis [37]. The mechanism by which a longer period of breastfeeding can reduce the risk of abdominal obesity in the long term is poorly understood. Visceral adipose tissue is more susceptible to reduction compared to subcutaneous adipose tissue because adipocytes of the epiploic and mesenteric adipose tissue, the main components of visceral fat, show greater metabolic activity and lipolytic sensitivity [41–43]. Subcutaneous and visceral adipocytes show differences in the expression of certain genes [44]; visceral adipocytes are characterised by higher lipolytic activity (significantly higher expression of the lipoprotein lipase gene) than subcutaneous adipocytes [45]. This may be important for fat mobilisation during lactation. Studies on rats [46] and humans [47, 48] have confirmed the preferential use of visceral fat over subcutaneous fat during lactogenesis. The CARDIA study showed that in a group of 910 participants, any duration of lactation was associated with significantly lower amounts of visceral fat [48]. The authors of that study did not, however, find a significant relationship between lactation and volume of subcutaneous, intermuscular, and total abdominal fat, after allowing for pre-pregnancy cardiometabolic factors. Kirkegaard et al., however, explain greater reduction of visceral than subcutaneous fat by the strong confounding effects of visceral adipose tissue before pregnancy [37]. If the greater amount of abdominal fat pre-pregnancy is associated with shorter breastfeeding duration, this may also explain the direct relationship between breastfeeding and waist circumference postpartum.

In the postmenopausal women we studied, those who gave birth to one child and breastfed for 7–12 months, had increased risk of abdominal obesity compared to those who did not breastfeed, which is contrary to expectations. This result may be due to the relatively small number of women who gave birth to only one child, but breastfed more than 6 months (3.19%). Our results did not reveal any significant association between breastfeeding and risk of excessive weight and obesity in premenopausal women. The values of adjusted ORs in this group, however, indicate a lower probability of lactating women to be overweight and have abdominal obesity, compared to those who have never breastfed. Our study also showed no correlation between the duration of lactation and percentage of body fat. Similarly, McClure et al., found increased visceral adipose tissue, measured by computed tomography 7 years postpartum among women who breastfed for > 3 months after the birth of each child, compared to those who breastfed longer [14]; they did not observe the same relationship for BMI and other body fat indices. Only Wiklund et al. found that women who breastfed their children for < 6 months had a higher body fat mass and %BF measured using dual-energy X-ray absorptiometry (DEXA), compared to those who breastfed for longer [35]. It is therefore possible that these associations are only revealed by more precise measurements of body fat, such as DEXA.

The main limitation of the study is that is was cross-sectional. The nutritional status of the participants before pregnancy was not known, nor was the amount of pregnancy weight gain. Therefore, these variables could not be included in the analysis as confounders. We also lacked detailed information on the intensity and regularity of breastfeeding. Some studies indicate that the effect of lactation on the risk of obesity may depend on whether the women breastfed their children exclusively or not [49]. The primary strength of the study is the large number of women included in the analysis (7500), taking into account the large number of confounding factors, including physical activity levels and nutritional factors, as well as conducting separate analyses according to parity and menopausal status.

## Conclusions

In women who gave birth to at least 2 children, breastfeeding was associated with a slightly lower risk of excessive weight and abdominal obesity after menopause, compared to women of the same parity who did not breastfeed at all. Breastfeeding may therefore have some beneficial, long-term effect on female fatness. Long-term studies are required in order to confirm the proposed association.

## Supplementary Information

**Additional file 1: Figure SM1**. Study flowchart.

**Additional file 2: Table SM1**. Multivariable logistic regression analysis for overweight and obesity in relation to breastfeeding duration in premenopausal women (unadjusted). **Table SM2**: Multivariable logistic regression analysis for overweight and obesity in relation to breastfeeding duration in postmenopausal women (unadjusted).

## Data Availability

The datasets used and analysed during the current study are available from the corresponding author on reasonable request.

## Abbreviations

%BF: Body Fat Percentage
BMI: Body Mass Index
CI: Confidence Intervals
LPA: Light Physical Activity
MET: Metabolic Equivalent of Task
MJ: Megajules
MVPA: Moderate to Vigorous Physical Activity
OR: Odds Ratio
PA: Physical Activity
WHtR: Waist to Height Ratio
