# Correction to: Breastfeeding history and the risk of overweight and obesity in middle-aged women

**DOI:** 10.1186/s12905-021-01378-2

**Published:** 2021-06-16

**Authors:** Elżbieta Cieśla, Ewa Stochmal, Stanisław Głuszek, Edyta Suliga

**Affiliations:** 1 Institute of Health Sciences, Medical College, Jan Kochanowski University, Kielce, Poland; 2 Institute of Medical Sciences, Medical College, Jan Kochanowski University, Kielce, Poland

## Correction to: BMC Women's Health (2021) 21:196 10.1186/s12905-021-01332-2

Following the publication of the original article [1], we were notified that the titles of the two Supplementary materials had been reversed.

The original article has been corrected.

## References

[1] Cieśla E (2021). Breastfeeding history and the risk of overweight and obesity in middle-aged women. BMC Women's Health.

