# Supplementary material for: Breastfeeding history and the risk of overweight and obesity in middle-aged women
Source: BMC Womens Health. 2021 May 11;21:196. doi: 10.1186/s12905-021-01332-2 (PMC8114504; doi:10.1186/s12905-021-01332-2)
Supplement: Supplementary file 1 — Additional file 1: Figure SM1. Study flowchart. [file 12905_2021_1332_MOESM1_ESM.docx]

**Breastfeeding history and the risk of overweight and obesity in middle-aged women**

Elżbieta Cieśla^1^, Ewa Stochmal^2^, Stanisław Głuszek^2^, Edyta Suliga^1^

^1^ - Institute of Health Sciences, Medical College, Jan Kochanowski University, Kielce, Poland

^2^ - Institute of Medical Sciences, Medical College, Jan Kochanowski University, Kielce, Poland

***** Corresponding author:

E-mail: eciesla@ujk.edu.pl

Table SM1. Multivariable logistic regression analysis for overweight and obesity in relation to breastfeeding dura­tion in premenopausal women (unadjusted).

| Parity | Breastfeeding status | BMI ≥ 25 kg/m^2^ | | %BF > 35% | | WHtR ≥ 0.5 | |
| --- | --- | --- | --- | --- | --- | --- | --- |
|  |  | OR; 95%CI | p | OR; 95%CI | p | OR; 95%CI | p |
| 1 child | Did not breastfeed | 1.00 |  | 1.00 |  | 1.00 |  |
|  | breastfeed 1-6 months | 1.00; 0.60-1.67 | 0.992 | 0.92; 0.55-1.54 | 0.747 | 0.92; 0.55-1.54 | 0.756 |
|  | 7-12 months | 0.95; 0.48-1.89 | 0.883 | 0.71; 0.35-1.44 | 0.347 | 0.90; 0.45-1.78 | 0.757 |
|  | > 12 months | 0.56; 0.26-1.21 | 0.142 | 0.45; 0.20-1.01 | 0.053 | 0.50; 0.23-1.09 | 0.082 |
| 2 children | Did not breastfeed | 1.00 |  | 1.00 |  | 1.00 |  |
|  | breastfeed 1-6 months | 0.80; 0.50-1.29 | 0.368 | 0.73; 0.46-1.16 | 0.182 | 0.89; 0.55-1.44 | 0.647 |
|  | 7-12 months | 0.93; 0.58-1.52 | 0.786 | 0.76; 0.47-1.23 | 0.265 | 0.87; 0.53-1.41 | 0.566 |
|  | > 12 months | 0.93; 0.57-1.50 | 0.766 | 0.81; 0.51-1.30 | 0.387 | 0.83; 0.51-1.34 | 0.450 |
| 3 and more children | Did not breastfeed | 1.00 |  | 1.00 |  | 1.00 |  |
|  | breastfeed 1-6 months | 0.43; 0.15-1.30 | 0.138 | 1.03; 0.42-2.52 | 0.951 | 0.53; 0.18-1.59 | 0.257 |
|  | 7-12 months | 0.47; 0.16-1.34 | 0.158 | 0.92; 0.39-2.15 | 0.846 | 0.65; 0.22-1.86 | 0.420 |
|  | > 12 months | 0.52; 0.19-1.42 | 0.205 | 1.08; 0.49-2.39 | 0.842 | 0.57; 0.21-1.56 | 0.276 |

BMI – body mass index; %BF – body fat percentage; WHtR – waist-to-hight ratio; OR – odds ratio; CI – confidence interval

Table SM2. Multivariable logistic regression analysis for overweight and obesity in relation to breastfeeding dura­tion in postmenopausal women (unadjusted).

| Parity | Breastfeeding status | BMI ≥ 25 kg/m^2^ | | %BF > 35% | | WHtR ≥ 0.5 | |
| --- | --- | --- | --- | --- | --- | --- | --- |
|  |  | OR; 95%CI | p | OR; 95%CI | p | OR; 95%CI | p |
| 1 child | Did not breastfeed | 1.00 |  | 1.00 |  | 1.00 |  |
|  | breastfeed 1-6 months | 1.12; 0.81-1.55 | 0.499 | 0.97; 0.71-1.32 | 0.845 | 1.20; 0.86-1.67 | 0.283 |
|  | 7-12 months | 1.29; 0.80-2.06 | 0.291 | 1.19; 0.76-1.86 | 0.448 | 1.47; 0.90-2.40 | 0.122 |
|  | > 12 months | 1.25; 0.66-2.35 | 0.490 | 0.88; 0.49-1.86 | 0.682 | 1.22; 0.64-2.32 | 0.548 |
| 2 children | Did not breastfeed | 1.00 |  | 1.00 |  | 1.00 |  |
|  | breastfeed 1-6 months | 0.76; 0.56-1.02 | 0.067 | 0.89; 0.68-1.16 | 0.374 | **0.65; 0.46-0.90** | **0.011** |
|  | 7-12 months | 0.79; 0.58-1.08 | 0.135 | 0.95; 0.72-1.25 | 0.702 | 0.77; 0.54-1.09 | 0.146 |
|  | > 12 months | 0.87; 0.63-1.21 | 0.420 | 1.01; 0.76-1.35 | 0.950 | **0.68; 0.47-0.97** | **0.032** |
| 3 and more children | Did not breastfeed | 1.00 |  | 1.00 |  | 1.00 |  |
|  | breastfeed 1-6 months | **0.42; 0.23-0.79** | **0.007** | 0.61; 0.37-1.02 | 0.060 | **0.50; 0.25-0.99** | **0.049** |
|  | 7-12 months | 0.58; 0.31-1.07 | 0.081 | 0.83; 0.51-1.35 | 0.446 | 0.68; 034-1.35 | 0.272 |
|  | > 12 months | 0.72; 0.40-1.30 | 0.275 | 1.00; 0.63-1.59 | 0.995 | 0.82; 0.43-1.58 | 0.562 |

BMI – body mass index; %BF – body fat percentage; WHtR – waist-to-hight ratio; OR – odds ratio; CI – confidence interval; **bold** indicate statistically significant results
