# Supplementary material for: Breastfeeding history and the risk of overweight and obesity in middle-aged women
Source: BMC Womens Health. 2021 May 11;21:196. doi: 10.1186/s12905-021-01332-2 (PMC8114504; doi:10.1186/s12905-021-01332-2)
Supplement: Supplementary file 2 — Additional file 2: Table SM1. Multivariable logistic regression analysis for overweight and obesity in relation to breastfeeding duration in premenopausal women (unadjusted). Table SM2: Multivariable logistic regression analysis for overweight and obesity in relation to breastfeeding duration in postmenopausal women (unadjusted). [file 12905_2021_1332_MOESM2_ESM.docx]

**Breastfeeding history and the risk of overweight and obesity in middle-aged women**

Elżbieta Cieśla^1^, Ewa Stochmal^2^, Stanisław Głuszek^2^, Edyta Suliga^1^

^1^ - Institute of Health Sciences, Medical College, Jan Kochanowski University, Kielce, Poland

^2^ - Institute of Medical Sciences, Medical College, Jan Kochanowski University, Kielce, Poland

***** Corresponding author:

E-mail: eciesla@ujk.edu.pl

Figure SM1. Study flowchart.
